# H-Ferritin Is Preferentially Incorporated by Human Erythroid Cells through Transferrin Receptor 1 in a Threshold-Dependent Manner

**DOI:** 10.1371/journal.pone.0139915

**Published:** 2015-10-06

**Authors:** Soichiro Sakamoto, Hiroshi Kawabata, Taro Masuda, Tatsuki Uchiyama, Chisaki Mizumoto, Katsuyuki Ohmori, H. Phillip Koeffler, Norimitsu Kadowaki, Akifumi Takaori-Kondo

**Affiliations:** 1 Department of Hematology and Oncology, Graduate School of Medicine, Kyoto University, Kyoto, Japan; 2 Laboratory of Food Quality Design and Development, Division of Agronomy and Horticultural Science, Graduate School of Agriculture, Kyoto University, Kyoto, Japan; 3 Department of Hematology, Japanese Red Cross Takatsuki Hospital, Takatsuki, Japan; 4 Department of Hematology and Immunology, Japanese Red Cross Otsu Hospital, Otsu, Japan; 5 Department of Clinical Laboratory, Kyoto University Hospital, Kyoto, Japan; 6 Division of Hematology and Oncology, Cedars-Sinai Medical Center, University of California Los Angeles, School of Medicine, Los Angeles, California, United States of America; 7 National University of Singapore, Singapore, Singapore; 8 Division of Endocrinology and Metabolism, Hematology, Rheumatology and Respiratory Medicine, Department of Internal Medicine, Graduate School of Medicine, Kagawa University, Takamatsu, Japan; CINVESTAV-IPN, MEXICO

## Abstract

Ferritin is an iron-storage protein composed of different ratios of 24 light (L) and heavy (H) subunits. The serum level of ferritin is a clinical marker of the body’s iron level. Transferrin receptor (TFR)1 is the receptor not only for transferrin but also for H-ferritin, but how it binds two different ligands and the blood cell types that preferentially incorporate H-ferritin remain unknown. To address these questions, we investigated hematopoietic cell-specific ferritin uptake by flow cytometry. Alexa Fluor 488-labeled H-ferritin was preferentially incorporated by erythroid cells among various hematopoietic cell lines examined, and was almost exclusively incorporated by bone marrow erythroblasts among human primary hematopoietic cells of various lineages. H-ferritin uptake by erythroid cells was strongly inhibited by unlabeled H-ferritin but was only partially inhibited by a large excess of holo-transferrin. On the other hand, internalization of labeled holo-transferrin by these cells was not inhibited by H-ferritin. Chinese hamster ovary cells lacking functional endogenous TFR1 but expressing human TFR1 with a mutated RGD sequence, which is required for transferrin binding, efficiently incorporated H-ferritin, indicating that TFR1 has distinct binding sites for H-ferritin and holo-transferrin. H-ferritin uptake by these cells required a threshold level of cell surface TFR1 expression, whereas there was no threshold for holo-transferrin uptake. The requirement for a threshold level of TFR1 expression can explain why among primary human hematopoietic cells, only erythroblasts efficiently take up H-ferritin.

## Introduction

Iron is essential for a variety of biological activities such as electron transfer, RNA synthesis, and oxygen delivery; however, excess iron can cause cellular damage by inducing the overproduction of reactive oxygen species [1]. Therefore, excess intracellular iron is stored in compartments in the form of ferritins, which are evolutionarily conserved from prokaryotes to plants and vertebrates [2]. In the latter, cytoplasmic ferritin forms spherical complexes composed of 24 H and L subunits; these are encoded by different genes and have approximately 50% amino acid sequence identity and similar 3-dimensional structures [3]. Each complex can store up to 4,500 ferric ions [4]. Only the H-subunit has ferroxidase activity for the conversion of iron incorporated into the ferritin shell from the ferrous to the ferric form [5]. The ratio of H and L subunits in ferritin heteropolymers varies depending on cell and tissue type; for example, the H and L subunits are more abundant in the heart and liver, respectively [6].

Ferritin is present in serum as well as in the cell. Serum ferritin is produced mainly by macrophages and hepatic cells through a non-canonical secretory pathway and its concentration correlates with the amount of iron stored in the body [7–9]. Ferritin expression increases in response to iron load as well as immune stimuli, and under certain inflammatory conditions, elevated serum ferritin levels reflect macrophage activation [10, 11]. The physiological functions of serum ferritin are unclear, although the H-ferritin homopolymer (HFt) was reported to inhibit normal hematopoiesis in vitro and in vivo, an effect that is linked to its ferroxidase activity [12–14], and can potentially suppress immune responses by modulating the functions of dendritic cells (DCs) and by activating regulatory T cells [15]. Whether serum ferritin leaks from iron-storing cells to perform these physiological functions is unknown.

Ferritin receptors are expressed by various cell types [16]. For example, human erythroid precursor cells possess specific receptors that bind and internalize HFt, a process that is regulated by intracellular iron status [17, 18]. T cell immunoglobulin and mucin domain (TIM)-2 and scavenger receptor class A member 5 are receptors for HFt and L-ferritin (LFt), respectively, in mice [19, 20]. In humans, there is no *Tim–2* ortholog although HFt receptors are expressed by various cell types [18, 21–23].

Recently, human transferrin receptor (TFR)1 was identified as a receptor for human HFt, despite transferrin (Tf) and ferritins having completely different molecular structures [24, 25]. The mechanism of how TFR1 mediates internalization of two different ligands, and the types of hematopoietic cell that preferentially incorporate HFt or LFt remain unknown. To address these questions, in this study we evaluated the capacity of various human blood cell types to incorporate ferritins as well as the mode of HFt uptake through TFR1 by flow cytometry.

## Materials and Methods

### Preparation of fluorescently labeled recombinant ferritin

Human recombinant ferritin H subunit was expressed in *Escherichia coli* strain BL21(DE3) (Novagen, Madison, WI, USA) and human ferritin L subunit was expressed using the pET system (Novagen) with the primer set 5'-AGC TCC CAG ATT CGT CAG AAT–3' and 5'-GCG AAG GAT CCT TAG TCG TGC TTG AGA GTG–3'; both proteins were purified as previously described [26, 27] and formed HFt and LFt homopolymers. The purity and integrity of the recombinant proteins were confirmed by sodium dodecyl sulfate gel electrophoresis under reducing and non-reducing conditions. Iron content of synthesized HFt and LFt, and cell lysates was measured using an atomic absorption spectrophotometer (AA–6800; Shimadzu, Kyoto, Japan) equipped with a graphite furnace atomizer. Protein concentrations were measured with the BioRad Protein Assay kit (Hercules, CA, USA) using bovine serum albumin as the standard. Endotoxin levels in the ferritin preparations were < 0.001%, as determined with the Limulus ES-II Single Test (Wako Pure Chemical Industries, Osaka, Japan). Recombinant ferritins and human holo-Tf were labeled with Alexa Fluor 488 (AF488; Life Technologies, Carlsbad, CA, USA) according to the manufacturer’s instructions. Iron-saturated holo-Tf (Calbiochem, San Diego, CA, USA) and AF488-labeled holo-Tf (Life Technologies) were used for comparisons.

### Cell culture

The human leukemia cell lines K562 [28], HEL [29], MEG–01 [30], CMK-11-5 [31], HL60 [32], NB4 [33], Jurkat [34], Reh [35], and THP–1 [36] were obtained from the Japanese Collection of Research Bioresources Cell Bank and maintained in Roswell Park Memorial Institute 1640 medium (Life Technologies) supplemented with 10% fetal bovine serum (FBS) at 37°C in a humidified atmosphere of 5% CO_2_, unless otherwise indicated. The erythropoietin-dependent erythroid cell line UT–7/Epo was kindly provided by Dr. Norio Komatsu and maintained in the same medium supplemented with 10% FBS and 1 U/ml recombinant human erythropoietin (Kyowa Hakko Kirin, Tokyo, Japan) [37]. The Chinese hamster ovary cell line CHO-TRVb, which lacks functional TFR1, was kindly provided by Dr. Timothy McGraw and maintained in Ham’s F12 nutrient mixture (Life Technologies) supplemented with 5% FBS [38].

### Patient samples

Peripheral blood and bone marrow (BM) cells from healthy donors (n = 5 and 3, respectively) and BM cells from myelodysplastic syndrome (MDS) patients (n = 4) were obtained with written, informed consent from the donors. The Institutional Review Board of the Graduate School of Medicine of Kyoto University approved the study protocol, which was carried out in accordance with the Helsinki Declaration.

### Confocal microscopy

K562 cells were incubated with 110 nM AF488-labeled recombinant human HFt homopolymer or 550 nM AF488-labeled human holo-Tf for 1 h at 37°C followed by incubation with Hoechst 33342 (Dojindo, Kumamoto, Japan). After washing with phosphate-buffered saline, cells were analyzed by confocal laser scanning fluorescence microscopy (Digital Eclipse C1; Nikon, Tokyo, Japan).

### Ligand binding and uptake assays

Cells were incubated with AF488-labeled ligands on ice (for the binding assay) or at 37°C (for the uptake assay) for 60 min, unless otherwise indicated, and analyzed using a FACSCalibur flow cytometer and CellQuest software (BD Biosciences, Franklin Lakes, NJ, USA). The ratio of mean fluorescence intensity (MFI) of AF488-labeled ligand-treated cells to that of untreated cells was used as an indicator of fluorescently labeled ligand binding or uptake. Competitive inhibition assays were performed using a 20-fold excess of unlabeled ligand unless otherwise indicated. Experiments were repeated at least three times to confirm reproducibility.

### Fluorescence-activated cell sorting

To determine the size of peripheral blood leukocyte and BM nucleated cell populations, cells were labeled with the following antibodies against human cell surface markers: anti-human cluster of differentiation (CD)235a-phycoerythrin (PE), CD14-PE-cyanin 5, CD16-PE, CD20-PE, CD203c-PE, CD3-PE-cyanin 5, CD4-PE-cyanin 5, CD4-PE-cyanin 7, CD8-PE-cyanin 5, and CD56-PE (all from BioLegend, San Diego, CA, USA); CD71-PE-cyanin 5, CD3-PE, and CD11c-PE (all from BD Biosciences); and CD141-allophycocyanin (Miltenyi Biotec, Bergisch Gladbach, Germany). The total lymphocyte population was determined from forward and side scatter, and CD4+ and CD8+ T cells, natural killer (NK) cells, and B cells were separated into CD3+/CD4+, CD3+/CD8+, CD56+/CD3−, and CD20+ populations, respectively. The monocyte population was determined from forward and side scatter and by CD14 expression. The granulocyte population was gated by forward and side scatter, and neutrophils and eosinophils were separated according to CD16 expression (CD16+ and CD16−, respectively). Basophils were isolated as the CD203c+ population. Plasmacytoid DCs, CD1c+ myeloid DCs, and CD141^high^ myeloid DCs were isolated from peripheral blood mononuclear cells on a FACSAriaII cell sorter as previously described [39]. Erythroid cells present in the BM cell population were gated according to CD235 and CD71 (TFR1) expression.

### Mutagenesis and cell transfection

We used a KOD-Plus-Mutagenesis kit (Toyobo, Osaka, Japan) to introduce point mutations into the human *TFR1* cDNA sequence to generate the R646H and G647A mutants. A FLAG-tag was also inserted at the 5'-end of the cDNAs, which were subcloned into pcDNA3.1 (Life Technologies). Stably transfected CHO-TRVb cell lines expressing either FLAG-tagged human wild-type or mutant TFR1 were established based on a published protocol [40].

### Statistical analysis

Data are presented as mean ± SE. Differences between test and control values were assessed with a paired t- test, and P < 0.05 was considered statistically significant.

## Results

### H-ferritin incorporation by K562 cells

Since human myeloid leukemia K562 cells express ferritin receptors [21], we used these cells to determine experimental conditions. The iron content of our recombinant human HFt and LFt was approximately 40 and 4 atoms, respectively, for each ferritin 24-mer.

**Fig 1:**
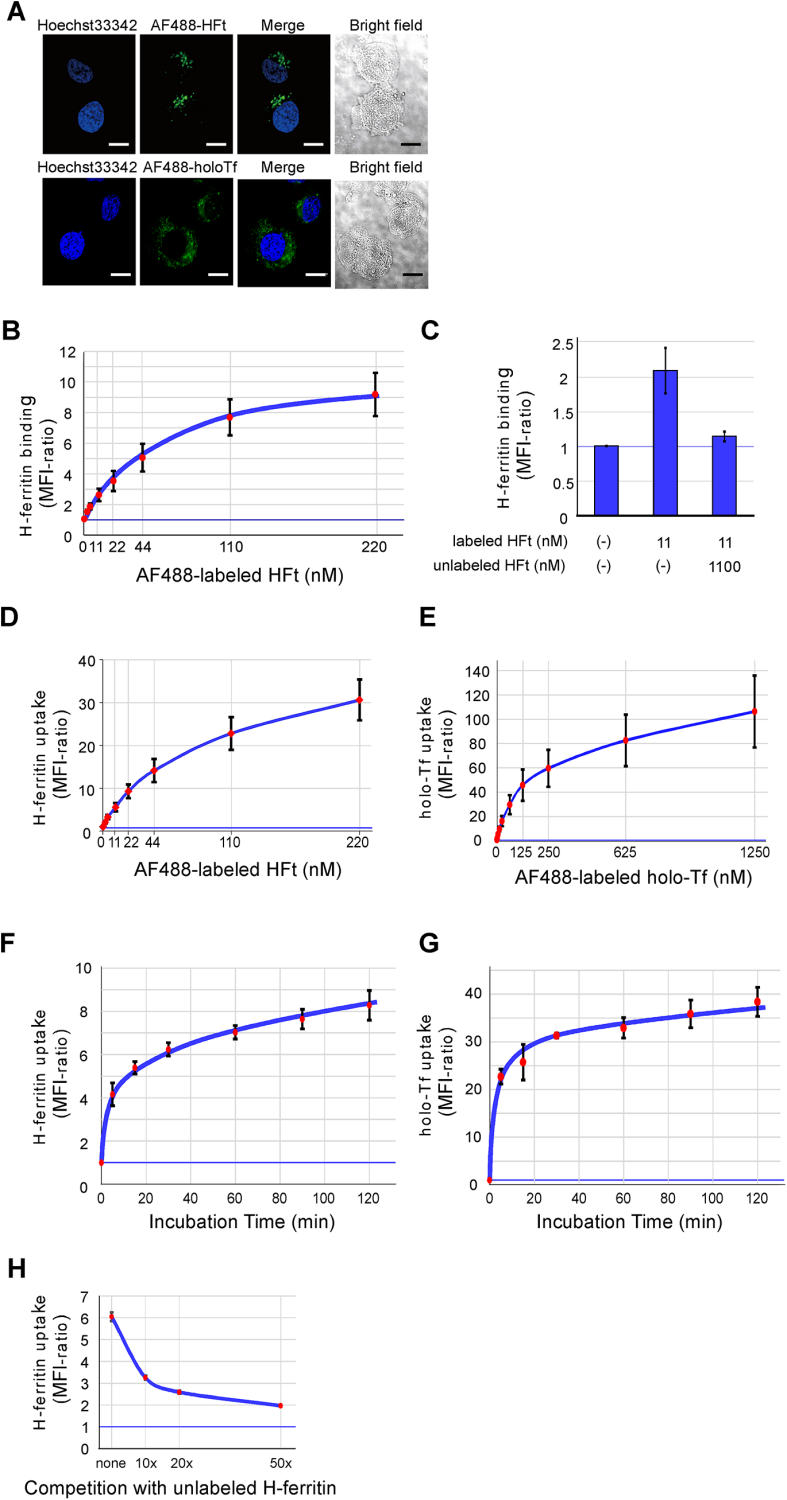
H-ferritin uptake by K562 cells. (A) Confocal micrographs of AF488-labeled H-ferritin (HFt) and holo-transferrin (Tf). Cells were incubated with 50 μg/ml (110 nM) AF488-labeled human recombinant HFt or 50 μg/ml (625 nM) holo-Tf for 1 h at 37°C. Images are representative results of three independent experiments. (B) Analysis of HFt binding to K562 cells. Cells were incubated with indicated concentrations of AF488-labeled HFt for 60 min on ice, and HFt binding was analyzed by flow cytometry. (C) Competitive inhibition of HFt uptake by K562 cells. Cells were incubated with 11 nM AF488-labeled ferritin in the presence or absence of a 100-fold excess of unlabeled HFt for 60 min on ice, followed by flow cytometric analysis. (D, E) Dose-dependency of HFt and holo-Tf uptake. Cells were incubated with indicated concentrations of AF488-labeled HFt or holo-Tf for 60 min at 37°C, and incorporation of these ligands was analyzed by flow cytometry. (F, G) Time-course of HFt and holo-Tf uptake by K562 cells. Cells were incubated with 11 nM AF488-labeled HFt or 62 nM holo-Tf for indicated times, and incorporation of these ligands was analyzed by flow cytometry. (H) Competition of AF488-labeled HFt uptake with unlabeled ligand. Cells were incubated with 11 nM AF488-labeled HFt and indicated concentrations of unlabeled HFt. (B–H) Data represent the means ± standard errors of three independent experiments.

We first evaluated the binding of HFt to cells, which were incubated with various concentrations of AF488-labeled HFt for 60 min at 0°C and analyzed by flow cytometry after fixation. HFt binding, as determined by the MFI ratio, increased in a dose-dependent manner, reaching near-saturation at 110 nM (Fig 1A). We chose a standard concentration of 11 nM (approximately 5,000 ng/ml) of AF488-labeled ferritin for subsequent experiments since it is an achievable concentration in sera from patients with severe iron overload [41, 42]. AF488-labeled HFt binding was almost completely inhibited by 100-fold excess of unlabeled HFt, indicating that this binding occurred through specific machinery (Fig 1B).

It was previously reported that HFt is rapidly incorporated into cells after binding to TFR1 and then transferred to endosomes and lysosomes; intracellular distribution of incorporated HFt differed from that of holo-Tf [24]. Consistent with this report, at 37°C, AF488-labeled HFt was rapidly incorporated by cells and visible as cytoplasmic puncta, in contrast to the more diffuse distribution of holo-Tf (Fig 1C). Thus, we confirmed that HFt does not remain at the plasma membrane but it is immediately internalized at 37°C.

We next examined the uptake of AF488-labeled ligands by these cells at 37°C. The MFI ratio increased in a dose-dependent manner; the increase was steep at concentrations <22 nM and more gradual at higher concentrations (up to 220 nM) (Fig 1D). For comparison, we also examined cellular holo-Tf uptake. Similar to HFt uptake, the incorporation increased in a dose-dependent manner with a steep increase at concentrations <62 nM, and a more gradual increase at higher concentrations (up to 1250 nM) (Fig 1E). The time course of HFt uptake showed that the MFI ratio increased sharply until 5 min (rapid phase), and which the increase was more gradual. Near-constant HFt uptake was observed between 30 min and 120 min (slow phase; Fig 1F). A similar pattern was observed in the time course of holo-Tf uptake (Fig 1G). Based on these results, we chose 60 min as the standard incubation time for ligand uptake assays. A competition assay was carried out using unlabeled ligand to determine whether HFt uptake was mediated by specific or non-specific mechanisms. Unlabeled HFt inhibited AF488-labeled HFt uptake in a concentration-dependent manner. In the presence of a 50-fold excess of unlabeled ligand, AF488-labeled HFt uptake was reduced to 20% of that in the absence of unlabeled ligand, indicating that approximately 80% of HFt uptake by K562 cells was mediated by a specific mechanism (Fig 1H).

### Ferritin uptake by hematopoietic cell lines

**Fig 2:**
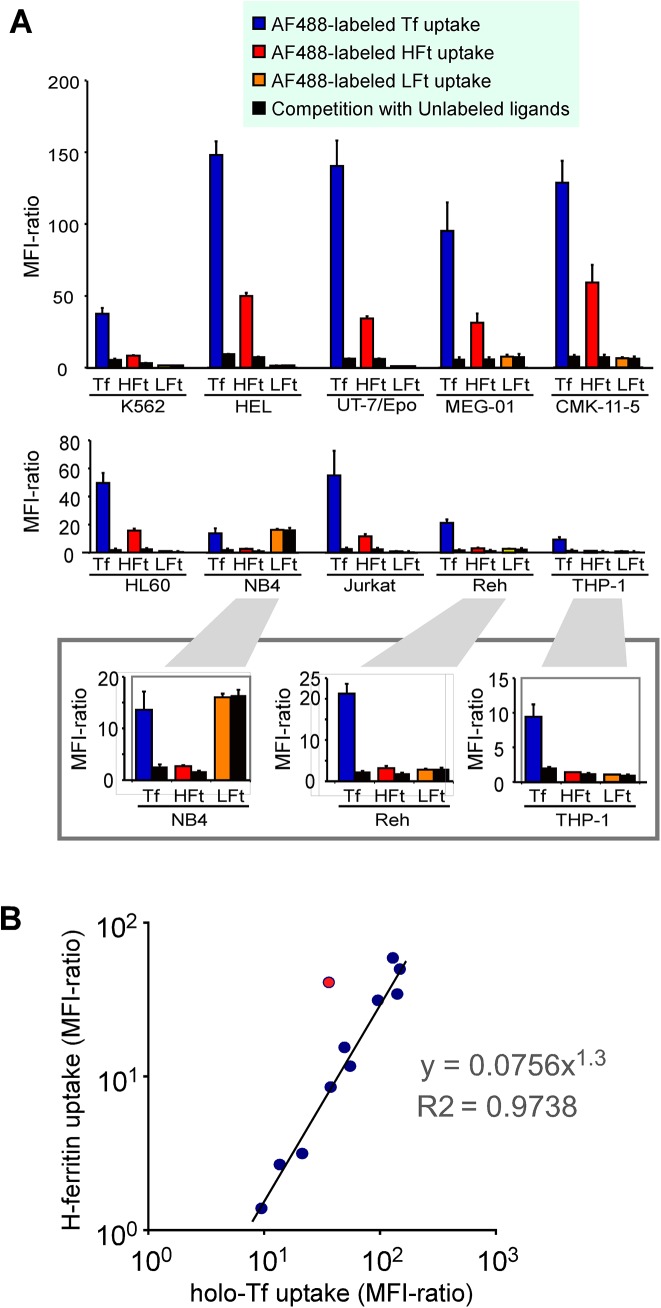
Analysis of H-ferritin and holo-transferrin uptake by hematopoietic cell lines. (A) Uptake of AF488-labeled human holo-transferrin (Tf), H-ferritin (HFt), and L-ferritin (LFt). Cells were incubated with AF488-labeled ferritins (11 nM) or Tf (62 nM) for 60 min and analyzed by flow cytometry. The MFI ratio was defined as the MFI of cells treated with fluorescence-labeled ligand divided by the MFI of untreated cells. Filled columns denote the results of competition experiments using a 20-fold excess of unlabeled cognate ligand. Graphs for NB4, Reh, and THP–1 cell lines are shown on a larger scale in the in the enclosed areas. Data represent the mean ± standard error of three independent experiments. (B) Relationship between HFt and holo-Tf uptake. Cell lines shown in (A) are shown as blue dots by their MFI ratios for HFt and holo-Tf uptake in a scatter plot (Spearman’s coefficient, ρ = 0.95; P < 0.0001). A red dot in this plot represents the position of human bone marrow erythroblasts shown in Fig 3B.

We evaluated the uptake of AF488-labeled human HFt and LFt by various human leukemia and lymphoma cell lines. Since TFR1 is the receptor for human HFt, we also examined the uptake of AF488-labeled human holo-Tf. All cell lines incorporated AF488-human holo-Tf, which was inhibited by unlabeled holo-Tf (Fig 2A). In contrast, K562, HEL, UT–7/EPO, MEG–01, CMK-11-5, HL60, and Jurkat cells preferentially incorporated HFt (MFI ratios of 9–59), whereas NB4 cells preferentially took up LFt (Fig 2A). Reh pre-B leukemia cells and THP–1 monocytic cells internalized modest amounts of HFt and LFt (MFI ratios < 4). Interestingly, strong HFt uptake (MFI ratios >30) was confined to cell lines exhibiting erythroid and megakaryocytic properties (i.e., HEL, UT–7/EPO, MEG–01, and CMK-11-5). HFt uptake by all cells was competitively but partially inhibited by a 20-fold excess of unlabeled ligand. In contrast, a large excess of unlabeled LFt did not prevent labeled LFt incorporation, suggesting a non-specific uptake mechanism. When we plotted these cell lines by their MFI-ratios of HFt uptake (y) and holo-Tf uptake (x), a linear relationship was observed (calculated best-fit curve, y = 0.0756x^1.3^, R2 = 0.9738) (Fig 2B), indicating that these ligands are internalized via a common pathway that is probably mediated by TFR1 [24]. These data also indicated that HFt uptake required much higher TFR1 expression than holo-Tf uptake.

### Ferritin uptake by primary human leukocytes

**Fig 3:**
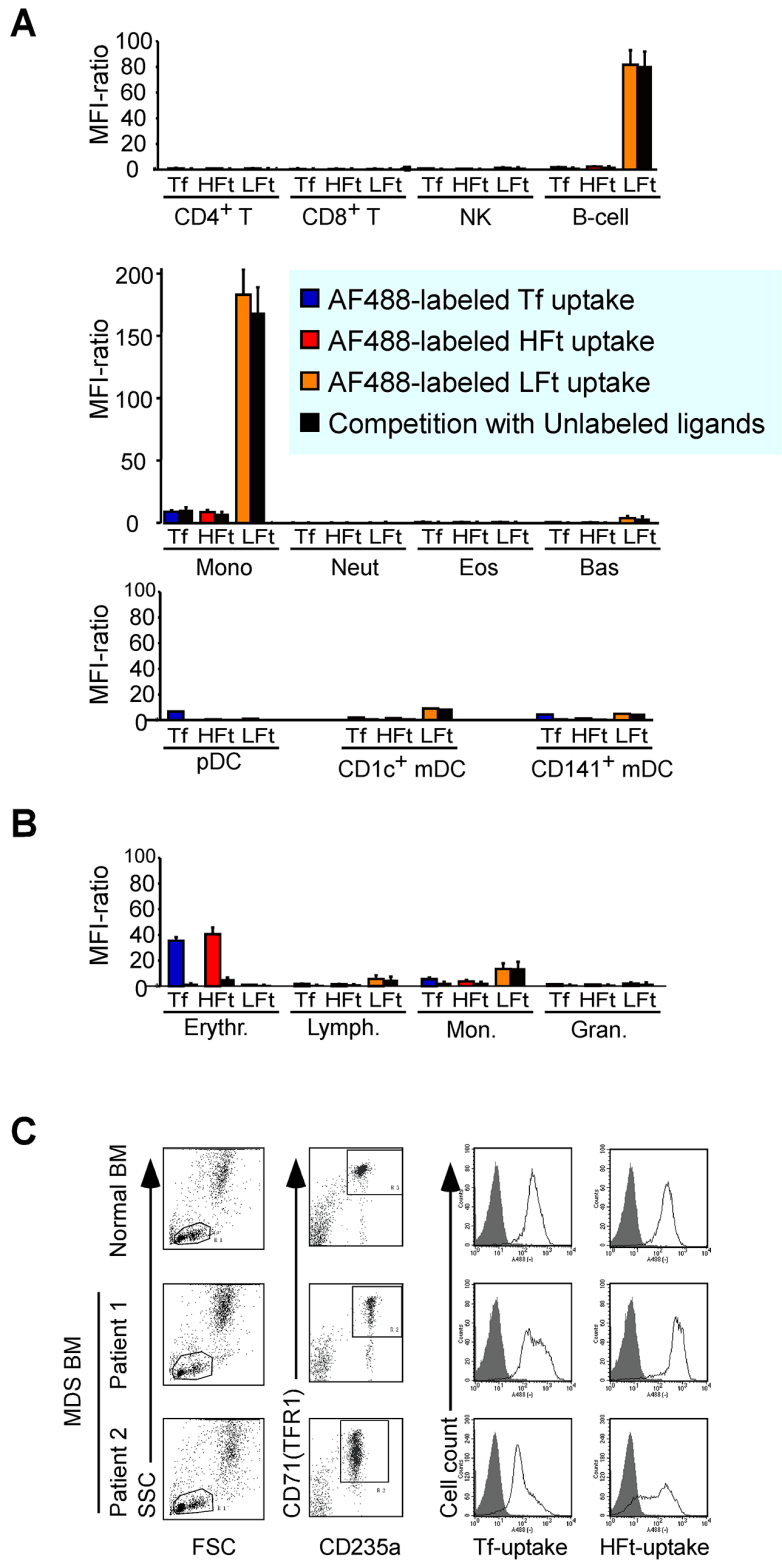
H-ferritin uptake by primary human hematopoietic cells. (A) Uptake of AF488-labeled human holo-transferrin (Tf), H-ferritin (HFt), and L-ferritin (LFt) by peripheral blood leukocytes. (B) Uptake of AF488-labeled Tf, HFt, and LFt by normal nucleated human BM cells. In (A) and (B), cells were incubated with AF488-labeled ferritins (11 nM) or Tf (62 nM) for 60 min and analyzed by flow cytometry. The MFI ratio was defined as the MFI of cells treated with fluorescence-labeled ligand divided by the MFI of untreated cells. Filled columns denote the results of competition experiments using a 20-fold excess of unlabeled cognate ligand. Data represent the mean ± standard error of three independent experiments. (C) Representative results of HFt uptake by erythroid cells from MDS patients. CD235+ erythroid cells from Patient 1 expressed close to normal levels of CD71 (TFR1) and efficiently incorporated HFt, whereas cells from Patient 2 expressed relatively low levels of TFR1 and incorporated less HFt as compared to cells from normal subjects. Solid lines represent AF488-labeled ligand uptake and shaded areas represent controls.

To determine whether the above observations are representative of ferritin uptake in a physiological context, we analyzed the incorporation of human holo-Tf, HFt, and LFt by various types of peripheral human blood cells harvested from healthy donors, which were incubated with AF488-labeled ligands and separated into CD4^+^ and CD8^+^ T cell, NK cell, B cell, monocyte, neutrophil, eosinophil, basophil, plasmacytoid DC, CD1c^+^ myeloid DC, and CD141^+^ myeloid DC populations based on forward and side scatter using antibodies against specific cell surface markers. Modest levels of holo-Tf were incorporated by monocytes, plasmacytoid DCs, and CD141^+^ myeloid DCs. The uptake of holo-Tf by DCs but not by monocytes was competitively inhibited by unlabeled ligand (Fig 3A). With the exception of monocytes, very little HFt was incorporated by these cells. Modest levels of AF488-labeled HFt were incorporated by monocytes, but the incorporation was not inhibited in the presence of excess unlabeled HFt. In contrast, high levels of LFt were taken up by B cells and monocytes, and to lesser degrees by CD1^+^ and CD141^+^ myeloid DCs; this was not blocked in the presence of excess unlabeled LFt, indicating that the uptake was non-specific (Fig 3A).

We also examined AF488-labeled ferritin internalization by normal human BM cells. Erythroblasts were separated as a CD235a^+^/CD71^+^ population, and based on side scatter, the remaining cells were separated into lymphocytes, monocytes, and granulocytic cells. Holo-Tf and HFt were taken up by erythroblasts; this was inhibited by a 20-fold excess of unlabeled ligand, suggesting a specific uptake mechanism (Fig 3B). HFt uptake was higher by erythroblasts than by HL60, Jurkat or K562 cells, which showed comparable levels of holo-Tf uptake (Fig 2A and the red dot in Fig 2B). Monocytes also incorporated holo-Tf and HFt, although their MFI ratios (5.6 and 3.8, respectively) were far lower than those of erythroblasts (36 and 41, respectively). Lymphocytes and granulocytic cells incorporated very little of either ligand (MFI ratios < 2).

We tested HFt uptake by BM cells from four MDS patients and found variable cell surface expression levels of TFR1 (Fig 3C). HFt uptake was reduced in samples from two patients with decreased levels of cell-surface TFR1 (Fig 3C, Patient 2).

### Effects of cellular iron status on H-ferritin uptake by erythroid cells

**Fig 4:**
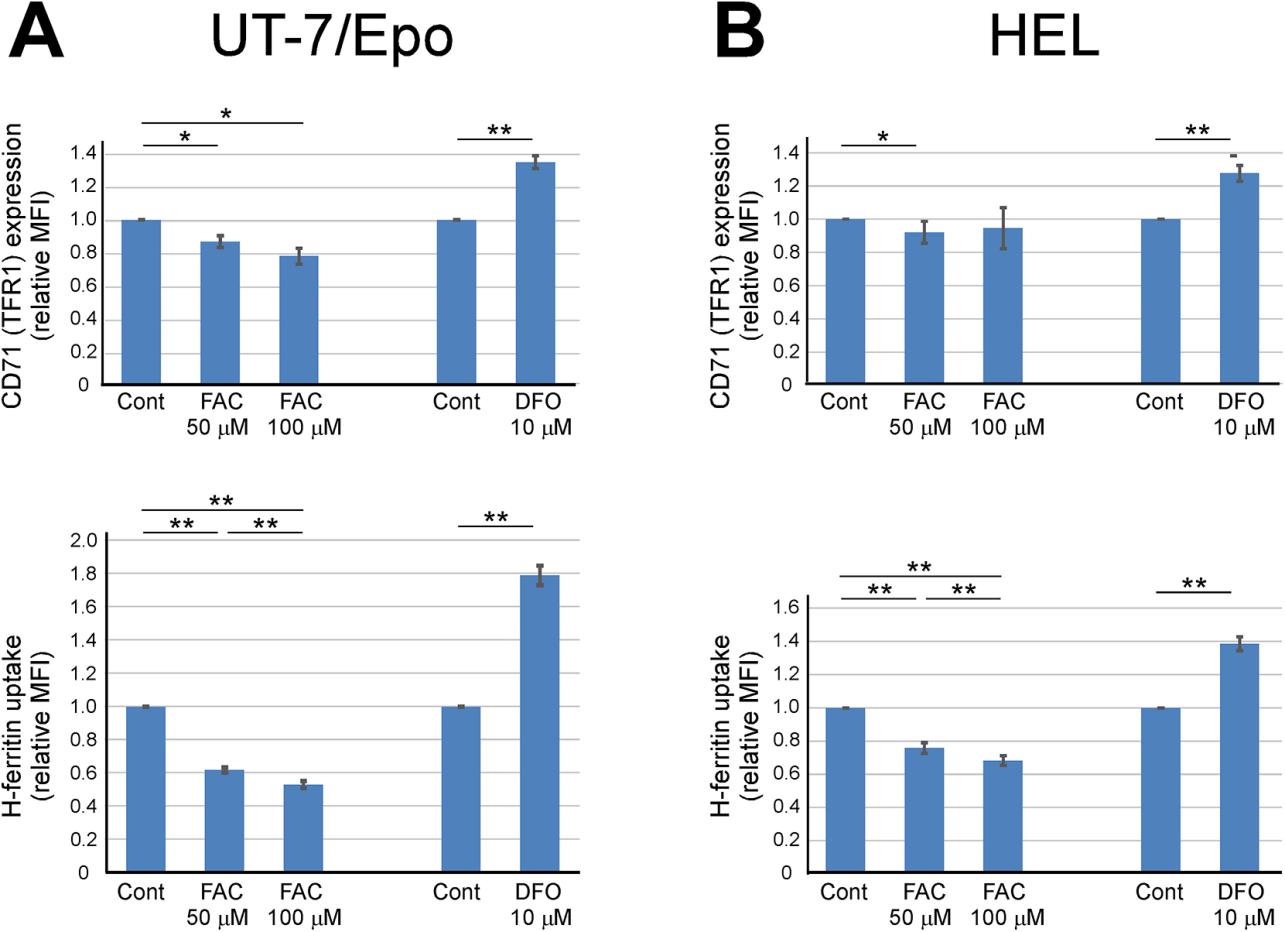
HFt uptake by erythroid cells in various iron statuses. (A, B) UT7/EPO and HEL cells were incubated with indicated concentrations of FAC or DFO for 24 h before flow cytometry analysis of CD71 (TFR1) expression (upper panels) and HFt (HFt) uptake (lower panels). MFI values relative to those of controls were calculated for each experiment; the results represent the mean ± standard error of at least three independent experiments. *P <0.05, **P <0.01; n.s., not significant.

To characterize the mechanism of HFt uptake by erythroid cells, we used UT–7/EPO erythroleukemia cell lines that exhibited enhanced HFt incorporation relative to K562 cells. Since TFR1 expression is downregulated in response to excess levels of iron and is upregulated when iron is chelated [43], we evaluated whether cellular iron status influences HFt uptake. Iron content of the UT–7/EPO cell lysate was approximately 3.2±0.3 μmol/g, and it became 6.0±0.3, 9.4±0.2 and 2.0±0.1 μmol/g after 24 h incubation with 50 μM ferric ammonium citrate (FAC), 100 μM FAC and 10 μM DFO, respectively. As expected, cell surface TFR1 (CD71) expression in UT–7/EPO was slightly reduced after a 24-h pre-incubation with FAC, and was increased by pre-incubation with desferrioxamine (DFO) (Fig 4A, upper panel). HFt uptake was reduced by pre-incubation with FAC in a dose-dependent manner and was enhanced by pre-incubation with DFO (Fig 4A, lower panel). Therefore, cellular iron status influences the incorporation of HFt by erythroid cells, providing evidence that TFR1 is an HFt receptor [24]. In these experiments, we noticed that changes in HFt uptake were larger than those in TFR1 expression. Similar results were obtained using HEL erythroleukemia cells (Fig 4B).

### Competition assays for H-ferritin and holo-transferrin uptake by erythroid cells

**Fig 5:**
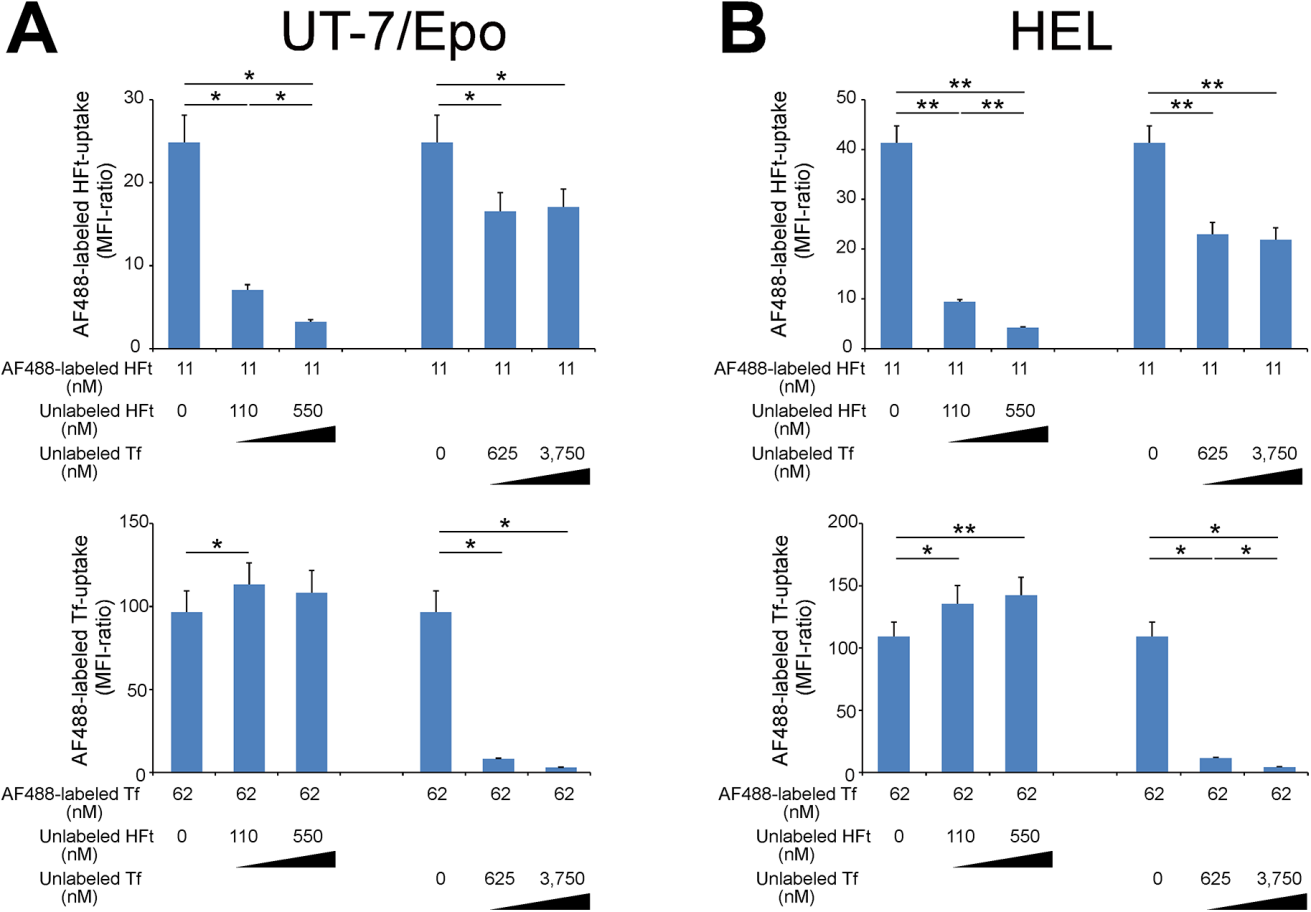
Effect of H-ferritin on holo-Tf uptake by erythroid cells. (A, B) Competition assays of the TFR1 ligands holo-transferrin (Tf) and H-ferritin (HFt). UT7/EPO (A) or HEL (B) cells were incubated with AF488-labeled holo-Tf or HFt along with the indicated concentrations of unlabeled ligand, and cellular uptake of AF488-labeled ligand was analyzed. Data represent the mean ± standard error of three independent experiments. *P <0.05, **P <0.01; n.s., not significant.

To determine whether holo-Tf and HFt bind to the same or to different sites on TFR1, we performed competition assays using unlabeled ligand. AF488-labeled HFt incorporation by UT–7/EPO cells was strongly inhibited by unlabeled HFt in a dose-dependent manner but was only partially inhibited by a large excess of unlabeled holo-Tf (Fig 5A, upper panel). In contrast, internalization of AF488-labeled human holo-Tf by the cells was effectively inhibited by unlabeled holo-Tf in a dose-dependent manner but was not inhibited by unlabeled HFt (Fig 5A, lower panel). Unexpectedly, unlabeled HFt enhanced holo-Tf uptake by cells. Similar results were obtained using HEL cells (Fig 5B). These results suggest that TFR1 mediates the uptake of Tf and HFt, which bind to different sites on TFR1.

### H-ferritin uptake by wild-type and mutant TFR1

**Fig 6:**
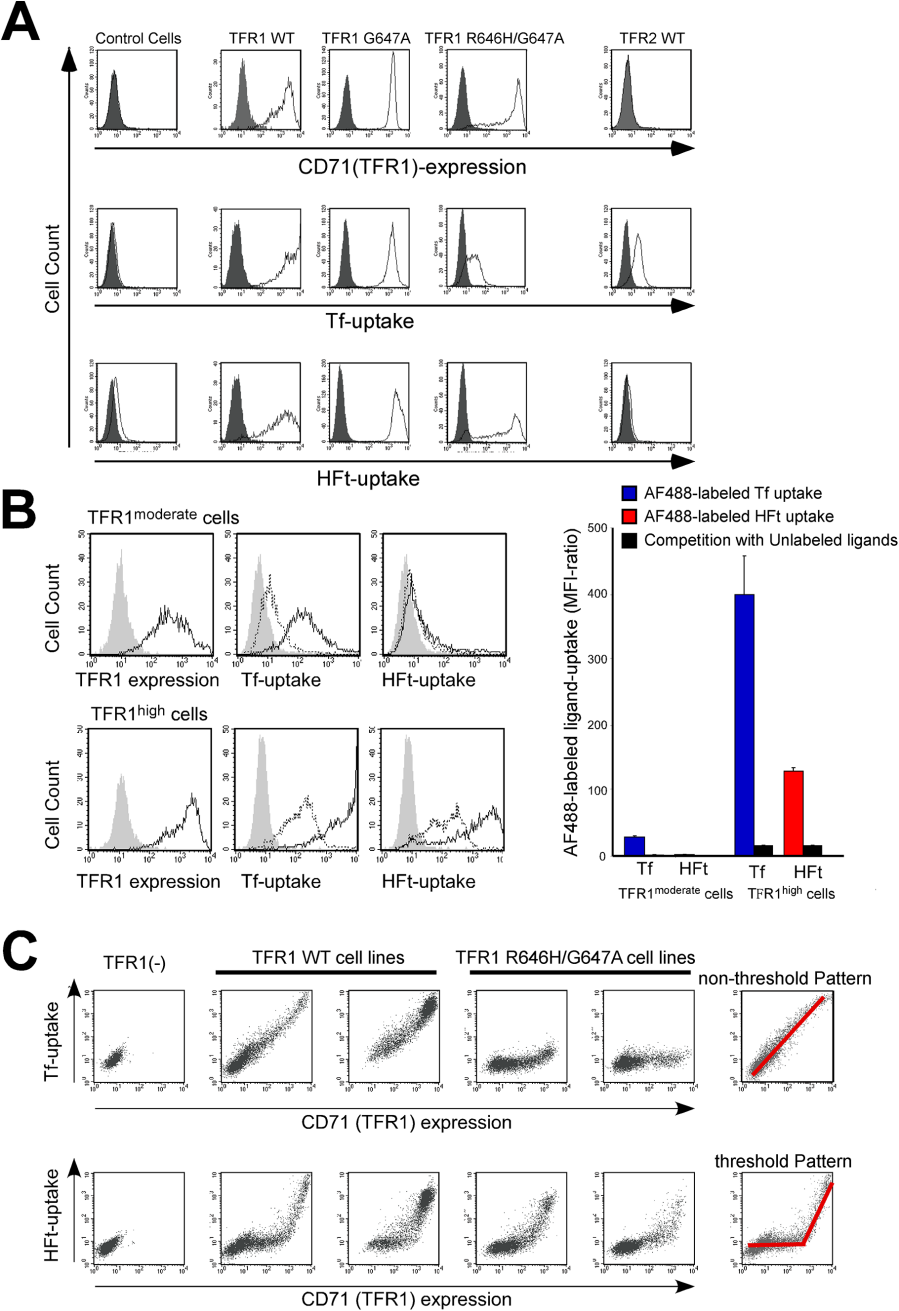
TFR1 expression levels required for H-ferritin uptake. (A) AF488-labeled H-ferritin (HFt) and holo-transferrin (Tf) uptake was evaluated in CHO-TRVb cells lacking endogenous TFR1 (control) and CHO-TRVb cells expressing either wild-type or mutant human TFR1 (R646H and R646H/647A) or wild-type human TFR2. Shaded areas represent isotype control experiments (CD71 expression) and untreated control experiments (Tf and HFt uptake). (B) AF488-labeled holo-Tf and HFt uptake by CHO-TRVb cells expressing moderate or high levels of human TFR1. Representative histograms are shown (left panels). Uptake of AF488-labeled ligand (solid lines), competition experiments using a 20-fold excess of unlabeled ligand (broken lines), and controls (shaded areas) are shown. Data shown in the right panel represent the mean ± standard error. (C) Relationship between CD71 expression and HFt uptake by CHO-TRVb cells expressing wild-type or mutant forms of human TFR1. Data from two cell lines expressing wild-type TFR1 and two expressing a mutated form of TFR1 (R646H/647A) are shown. Right panels show a schematic representation of two different patterns of ligand uptake.

We established CHO-TRVb cell lines that express wild-type or mutant forms of human TFR1 and analyzed their uptake of holo-Tf and H- and L-ferritins. We introduced mutations in the RGD sequence (R646H/G647A) of TFR1, which is essential for binding Tf [44]. Consistent with a previous report, TFR1-deficient CHO-TRVb cells did not incorporate HFt, in contrast to CHO-TRVb cells stably transfected with wild-type human TFR1 (Fig 6A, left) [24]. CHO-TRVb cells expressing TFR2 also failed to incorporate HFt (Fig 6A, right). The presence of R646H/G647A mutations in TFR1 markedly reduced holo-Tf but not HFt uptake (Fig 6A, third and fourth panels from the left).

CHO-TRVb cell lines expressing different levels of TFR1 revealed that HFt incorporation was positively correlated with increased TFR1 expression (Fig 6B). The TFR1^high^ cells incorporated a large amount of A488-labeled HFt, whereas only modest incorporation was observed for TFR1^moderate^ cells, indicating that HFt uptake required high levels of TFR1 expression. Uptake of AF488-labeled HFt was competitively inhibited by unlabeled HFt, confirming that uptake occurred through specific machinery (Fig 6B). In these wild-type TFR1-expressing CHO-TRVb cell clones, the cell-surface expression of TFR1 in individual cells was highly variable, and was linearly related to holo-Tf uptake (Fig 6C, upper panels). In contrast, the correlation between TFR1 and HFt uptake was linear only above a threshold level of TFR1 expression (Fig 6C, lower panels). CHO-TRVb cells expressing mutant TFR1 (R646H/G647A) did not efficiently incorporate holo-Tf but their uptake of HFt was similar to that of cells expressing wild-type TFR1 (Fig 6C, fourth and fifth panels from the left).

## Discussion

Since the 1980s, several reports have demonstrated that different types of human blood cell have specific HFt binding sites [18, 21–23], but the specific HFt receptor in these cells were not identified for a long time. Recently, human TFR1 was identified as an HFt receptor that is quickly transported to endosomes and lysosomes following TFR1-mediated endocytosis [24]. Since the binding of HFt to TFR1 was previously investigated [24], in the current study, we focused on the mechanism of HFt uptake by cells. Analyzing ligand uptake—which includes ligand binding to the plasma membrane, endocytosis, and intracellular trafficking—is important for understanding the physiological roles of the ligand. We established a quantitative flow cytometry-based method for measuring ligand uptake using AF488 and confirmed that holo-Tf and HFt are incorporated by human cells through a TFR1-medited mechanism (Fig 1).

TFR1 is highly expressed in cells that require high levels of iron such as erythroid precursors, rapidly proliferating cells, and cancer cells [45]. Accordingly, various human leukemia and lymphoma cell lines—especially erythroid leukemia cell lines—incorporate AF488-labeled HFt (Fig 2). We showed here that among primary human hematopoietic cells, erythroid precursor cells expressing relatively high levels of TFR1 preferentially and specifically take up HFt (Fig 3). We detected very low levels of HFt incorporatin by immune cells such as lymphocytes and dendritic cells (Fig 3), but previous studies have reported that ferritin has immunosuppressive effects in vitro as well as in patients [15, 46, 47], indicating that immune cells also express an HFt receptor. Cell surface TFR1 expression is low in steady-state lymphocytes; but once activated, these cells express high levels of TFR1 [48]. These activated lymphocytes are presumed to incorporate HFt, which is supported by a previous report demonstrating that lymphoid cells upregulated HFt receptor expression in the presence of phytohaemagglutinin, and that HFt inhibited the growth of phytohaemagglutinin-stimulated lymphocytes [22].

Dysplastic erythroid cells in MDS patients were reported to express relatively low levels of TFR1 as compared to normal erythroid precursors [49]. Consistent with these observations, we found that cell-surface TFR1 expression was lower in erythroid cells from two of our MDS patients than in normal erythroid cells. Given that a threshold level of TFR1 expression is necessary for HFt uptake, dysplastic erythroid cells expressing a low level of TFR1 may not efficiently incorporate HFt (Fig 3C).

In the current study, we also investigated the difference between HFt uptake and holo-Tf uptake through TFR1, and demonstrated that HFt uptake requires a threshold level of cell surface TFR1 expression, in contrast to Tf uptake for which there is no threshold (Fig 6C). This was supported by an analysis of the relationship between holo-Tf and HFt uptake by various hematopoietic cell lines; the calculated best fit curve indicated that much higher TFR1 expression was required for HFt uptake than for holo-Tf uptake (Fig 2B). The requirement for a threshold level of TFR1 expression can explain why only certain cell types such as erythroid cells efficiently take up HFt, while other cell types can efficiently incorporate holo-Tf but not HFt (Figs 2 and 3), and why changes in HFt uptake by erythroid cells were greater than changes in TFR1 expression (Fig 4). The threshold-dependent pattern of HFt uptake may suggest that more than one TFR1 complex is required for HFt incorporation. Primary human erythroblasts from bone marrow showed greater efficiency of HFt uptake relative to hematopoietic cell lines which showed comparable levels of holo-Tf uptake (Fig 2A and the red dot in Fig 2B). The reason of this phenomenon is unknown, but bone marrow erythroblasts may have different distribution patterns of TFR1 on the plasma membrane, they may have a co-receptor for facilitating HFt uptake, or they may possess efficient intracellular trafficking machineries for incorporated HFt.

TFR1 forms a homodimeric complex at the cell surface that can interact with two Tfs. The results of our competition assays showed that HFt and Tf do not compete for TFR1-mediated cellular uptake, indicating that the TFR1 complex has distinct binding sites for HFt and Tf. This is supported by the fact that the RGD sequence—which is essential for holo-Tf uptake—was not required for HFt incorporation by cells expressing TFR1 (Fig 6, panels A and C). Unexpectedly, unlabeled HFt rather enhanced incorporation of AF488-labeled Tf by HEL cells, suggesting that HFt facilitates holo-Tf uptake through undetermined mechanisms (Fig 5A).

There were limitations to the present study. Firstly, we used recombinant proteins synthesized in bacteria that were not subjected to posttranslational modifications such as glycosylation, which may have influenced the results. Secondly, we analyzed HFt uptake only in vitro. In mice, TIM2 serves as a HFt receptor, but there are no *Tim2* orthologs in the human genome [50]. Therefore, mechanisms of HFt uptake in mice may not reflect human serum ferritin activity. Thirdly, we are not able to separate binding and intracellular trafficking by flow cytometry in the current study. The time course experiments of HFt and holo-Tf uptake shown in Fig 1, panels E and F indicated that ligand uptake can be separated at least into 3 phases; the rapid phase, gradually retarding phase, and the slow phase in which the MFI-increasing ratio becomes constant. We assume that the rapid phase represents the ligand binding and the early steps of endocytosis, and the following slow phase represents the rate-limiting steps in the intracellular trafficking. Such time course experiments may be useful to dissect these steps and to separately estimate the efficacies of these two phases of the ligand uptake process.

It was recently demonstrated that endogenous cytoplasmic HFt is transported to autophagosomes through a nuclear receptor coactivator 4-mediated mechanism [51, 52]. Serum HFt endocytosed by TFR1 may be transported to lysosomes via a similar mechanism given that the intracellular domain of TFR1 has been shown to interact with γ-aminobutyric acid A receptor [53], which is a potential binding partner of nuclear receptor coactivator 4 [51]. Cells may utilize iron released by degraded ferritin, but it is unlikely that serum ferritin is a major source of cellular iron [54].

In conclusion, we showed here that among human hematologic cell types, erythroid cells preferentially incorporate HFt through a TFR1-mediated mechanism. We also showed that TFR1 has distinct binding sites for HFt and Tf. Moreover, we showed that cellular uptake of Tf and HFt by TFR1 differ in that only the latter depends on a threshold of level of ligand. Further studies are required to clarify the fate of HFt after internalization, and the roles of HFt in erythropoiesis in physiological and iron overloaded conditions.
